# Optimal gestational weight gain for Chinese women - analysis from a longitudinal cohort with childhood follow-up

**DOI:** 10.1016/j.lanwpc.2021.100190

**Published:** 2021-07-06

**Authors:** Yuanying He, Claudia Ha-Ting Tam, Lai Yuk Yuen, Patrick M. Catalano, Ronald Ching-Wan Ma, Wing Hung Tam

**Affiliations:** 1 Department of Obstetrics and Gynaecology, The Chinese University of Hong Kong, Prince of Wales Hospital, Hong Kong Special Administrative Region, China; 2 Department of Medicine and Therapeutics, The Chinese University of Hong Kong, Prince of Wales Hospital, Hong Kong Special Administrative Region, China; 3 Maternal Infant Research Institute, Tufts University School of Medicine, Tufts Medical Center, Boston, MA, USA.; 4 Hong Kong Institute of Diabetes and Obesity, The Chinese University of Hong Kong, Hong Kong Special Administrative Region, China; 5 Li Ka Shing Institute of Health Sciences, The Chinese University of Hong Kong, Shatin, Hong Kong Special Administrative Region, China

**Keywords:** Gestational weight gain (GWG), Reference ranges, Hyperglycemia and Adverse Pregnancy Outcomes (HAPO) study, Longitudinal cohort, Neonatal adiposity, Birth weight, Childhood cardiometabolic risk factors

## Abstract

**Background:**

Maternal gestational weight gain (GWG) influences not only on pregnancy outcome but also impacts on mothers’ and children's long-term health. However, there is no consensus on recommendations of optimal GWG in Asians or the Chinese population.

**Methods:**

We performed a secondary analysis of the birth outcome of Chinese women who had joined the “Hyperglycemia and Adverse Pregnancy Outcome” study in Hong Kong and their children's cardiometabolic risk at 7-year of age. Optimal ranges of GWG were derived from models based on the probabilities of small for gestational age and large for gestational age (model 1), lean and fat infants (model 2) and the integration of model 1 and 2 (model 3), and were compared with that recommended by the Institute of Medicine (IOM) on children's cardiometabolic risk.

**Findings:**

GWG range derived from model 2 is associated with 8 cardiometabolic risk factors, while that from models 1 and 3 are associated with 1 and 7 of them respectively. Mothers whose GWG lie within the recommended range increases from 40.8% according to the IOM recommendation to 50.2% according to that derived from model 2.

**Interpretation:**

Optimal GWG derived from model 2 (i.e. 14.0-18.5 kg, 9.0-16.5 kg and 5.0-11.0 kg for underweight, normal weight and overweight Chinese women, respectively) appeared to be associated with the lowest cardiometabolic risk in the offspring.

**Funding:**

General Research Fund of the Research Grants Council of the Hong Kong SAR, China (grants CUHK 473408 and, in part, CUHK 471713).

**Research in context**

**Evidence before this study**

• Optimal gestational weight gain (GWG) is an important factor to the pregnancy outcome and the long-term health of the offspring.
• Most of the previous studies evaluate the risk of small for gestational age (SGA) and large for gestational age (LGA) to determine the optimal GWG.
• The most used reference was that recommended by the Institute of Medicine (IOM) but was predominantly for the Caucasian population.
• There is a paucity of literature in the optimal GWG for Asian or Chinese population.

**Added value of this study**

• Compared with optimal GWG based on the infants’ SGA and LGA, optimal maternal GWG derived from infants’ adiposity appears most discriminative with the association of children's future cardiometabolic risk.
• Infants’ fat mass and fat-free mass both increase at different rates with their own birth weight and with maternal GWG related to the mothers’ pre-pregnancy BMI, so that the percentage of body fat increases with both infants’ birth weight and maternal GWG.
• More women would fall into optimal GWG based on model derived from infants’ adiposity (50.2%) than that based on the IOM recommendation (40.8%).
• Optimal GWG ranges for multiparity appeared to be lower than those for the nulliparity as well as for the whole population.

**Implications of all the available evidence**

• Our study provides a reference on the methodology for future study to determine optimal GWG in the Chinese or Asian population.
• Neonatal body fat percentage can now be reliably estimated from routine anthropometric measurements at birth so it is feasible to include adiposity as an outcome index to determine optimal GWG.
• The optimal GWG may differ between primigravida and multiparity so future studies on should consider parity as a factor of GWG.

Alt-text: Unlabelled box

## 1 Introduction

Maternal gestational weight gain (GWG) influences pregnancy outcomes, as well as mother's and offspring's long-term health. Excessive GWG increases the mother's risk of caesarean delivery, gestational hypertension and gestational diabetes mellitus (GDM), and the baby's risk of being large for gestational age (LGA) and macrosomia. Excessive GWG also increases the risk of postpartum weight retention and type 2 DM. [2] On the other hand, inadequate GWG, especially among those underweight mothers, increases the risks of preterm birth, small for gestational age (SGA) and low birth weight (LBW). [1] Furthermore, GWG, either inadequate or excessive, can also have negative effects on the offspring's blood pressure, adiposity, insulin resistance and pancreatic beta cell function. [3]

Whilst there are still controversies about the practice of routine weighing in pregnancy and the reference on optimal GWG, the recommendations from the Institute of Medicine (IOM) last revised in 2009 is the most commonly used reference for the Caucasian population. However, the IOM guidelines are based on data from USA-dwelling, Caucasian and Black women that may not be generalizable to women from Asia. In fact, studies from the Asian continent suggested that Chinese and Japanese mothers should have GWG less than that proposed by the IOM. [4,5] Compared with Caucasians, Asians also have greater body fat mass, lower lean body mass and higher cardiometabolic risks at the similar body mass indeces (BMI). [6,7] The categorisation of underweight, normal weight, overweight and obesity based on BMI are different between Caucasian and Asian women. There were several studies in Japanese, Korean and Vietnamese recommending different ethnicity-specific guidelines on GWG. [5,8,9] Hence, GWG guidelines for Asian women should be considered separately and differently.

Most of the previous studies derived optimal GWG based on infants’ birth weight that result in the lowest rates of SGA and LGA at delivery. However, other than the birth weight, GWG also impacts on offspring's body fat composition, [10,11] adiposity at birth can be perpetuated from early childhood, [3] through adolescence [12] into adulthood. [13] The aim of the present study is to examine the role of applying infants’ body fat mass in deriving an optimal GWG for the Chinese population. We had previously reported that children whose mother had GWG outside the ranges recommended by the IOM had greater cardiometabolic risks at seven years of age. [3] In this study, we also investigate how the optimal GWG derived from our methodology compared with that of the IOM in predicting offspring's cardiometabolic risk at early childhood.

## 2 Methods

### 2.1 Study design

This is a secondary analysis of data obtained from the mother-child dyads who participated in the original “Hyperglycemia and Adverse Pregnancy Outcome (HAPO)” study in 2000-2006, and from those who also participated the follow-up study during 2009-2013 in Hong Kong. In the original HAPO study, 1667 pregnant women with a singleton pregnancy underwent a 75 g oral glucose tolerance test (OGTT) at 24–32 weeks’ gestation at the Hong Kong study centre. All the participants and clinicians were blinded from the OGTT result, but mothers whose glucose levels at the OGTT or the random glucose levels at late gestation exceeded the pre-determined safety range were treated accordingly and were excluded from further study. [14] Meanwhile, women who were non-Chinese, delivered a preterm (before 37 weeks) baby or a stillbirth were excluded for the secondary analysis. At the follow-up study, we assessed 882 children at seven years of age by performing anthropometric, blood pressure measurement and a five-point OGTT with insulin levels at the assessment. The details of the study design were described previously elsewhere. [3,15] The flow chart of the study is shown in the supplementary figure 1.

Pre-pregnant weight was obtained by participant’ recall at the first antenatal visit and at recruitment of the original HAPO study. Maternal height was measured at the first antenatal visit. Maternal weight just before delivery was measured in the antenatal ward upon admission in early labour. The gestational weight gain was obtained from the data set of the HAPO study and verified from the electronic data captured into the hospital computerised system.

We adopted the BMI categorization endorsed by the National Health and Family Planning Commission of China for Chinese population. [16] Mothers were classified according to their pre-pregnancy BMI as underweight (BMI <18.5 kg/m^2^), normal weight (BMI 18.5 – 23.9 kg/m^2^), overweight (BMI 24.0 – 27.9 kg/m^2^), and obese (BMI≥28 kg/m^2^).

Neonatal measurements were obtained in a standardized method by trained research staff within 72 hours of delivery as previously described in the original HAPO study. [17] The anthropometric measurements including weight, length, and skinfold thickness at flank were obtained in duplicate. If the results differed by more than a pre-specified amount (i.e. >10 g for weight, >0.5 cm for length, or >0.5 mm for skin folds), a third was done. ^17^ For these analyses, the average of the two measurements was used unless a third measurement was taken. Birth weight was obtained without diaper using a calibrated electronic scale. Flank skinfold thickness was measured with calipers (Harpenden, Baty, U.K.) on the neonate's left side, just above the iliac crest on a diagonal fold on the mid-axillary line.

LBW and macrosomia were defined when the infant's birth weight was below 2500 g and above 4000g, respectively. In the present study, SGA and LGA were defined with reference to the data obtained from a territory-wide study conducted between 1998 and 2001 in Hong Kong that included ten-thousand Chinese infants to establish gestational age-specific birth weights for boys and girls. [18] Infants were classified as SGA and LGA when the birth weight was below the 10^th^ percentile and above the 90^th^ percentile of the standard growth curve respectively. Infant's body fat mass was estimated with the equation: 0.39055 (birth weight) + 0.0453 (flank skinfold) - 0.03237 (length) + 0.54657 as previously used in the HAPO study. [17,19] Fat-free mass was calculated by subtracting fat mass from birth weight. Body fat mass was adjusted for gestational age (GA) and gender by using linear regression before further analyses. Neonates were classified as fat and lean when the standardized fat mass was greater than 90^th^ percentile and less than 10^th^ percentile, respectively. The percentage of body fat mass, i.e. fat mass divided by birth weight, was used as a surrogate marker for body fat composition to quantify neonatal adiposity. Data were available for the estimation of fat mass in 1392 (92.9%) neonates. The equation to estimate fat mass has been validated against PEA POD and with reference to equations used in Asians populations. [20]

### 2.2 Statistical analysis

Categorical variables were compared by using Chi-Square test or Fisher's exact test. Normally distributed continuous variables were expressed as mean ± SD and compared using one-way ANOVA. Non-normally distributed continuous variables were expressed as median (IQR) and compared by using Kruskal-Wallis tests. Post-hoc pairwise comparison was performed when the null hypothesis was rejected. Binary regression models, both “logit” and “complementary log-log” links, were used to assess the associations between the odds of SGA, LGA, fat and lean at birth with maternal GWG among mothers who were underweight, normal weight and overweight; there were too few obese mothers (n=33) for the statistical analysis. The link with a lower Akaike information criterion (AIC) value was regarded as a better model, and was used for the development of the reference ranges for optimal GWG. Odds ratios (ORs) with 95% confidence intervals (CIs) were obtained after adjusting for maternal age, parity, gestational hypertension/preeclampsia, smoking status in pregnancy and maternal glycaemic status in pregnancy [represented by the area under the curve of glucose levels (AUC _glu_) at OGTT during pregnancy]. The optimal GWG in each subgroup was derived based on three models: model 1 by birth weight (targeted to the lowest sum of probabilities of SGA and LGA); model 2 by neonatal adiposity (targeted to the lowest sum of probabilities of fat and lean infants); and model 3 by integrating models 1 & 2 (targeted to the lowest sum of probabilities of SGA, LGA, fat and lean infants). Equations to calculate the individual probability (equation 1 and 2) and sum of probabilities (equation 3 to 5) were described in the supplementary note 1. The lower and upper bounds of the optimal GWGs were the points at which 1% increase in the sum of probabilities from its lowest value were observed [5] (Supplementary figure 2). The proposed reference ranges further corrected to the nearest 0.5 kg where the risks of women lie with in the ranges had no more than 1% higher than the lowest risk. Additionally, nulliparity was treated as a dichotomous covariate. Risks of infants born from nulliparous women versus multiparous women were calculated, respectively, as a sensitivity analysis.

We applied the optimal ranges of GWG obtained from model 1 to model 3 to assess children's cardiometabolic risk factors at seven years of age, which include adiposity traits (i.e. BMI, waist circumference, sum of skinfold thickness at four sites, namely, biceps, triceps, subscapular and suprailiac), blood pressure [i.e. systolic and diastolic blood pressures (SBP and DBP)], glucose and insulin levels [i.e. fasting plasma glucose and insulin (FPG and FPI), 2 h glucose and insulin, as well as beta cell function and insulin sensitivity [i.e. HOMA-β, insulinogenic index, HOMA-IR, Matsuda insulin sensitivity index (ISI) and oral disposition index (ODI)] and compared them with the GWG recommended by the IOM using the analyses we described previously elsewhere. [3] All traits were adjusted for maternal age at the expected date of confinement (EDC), GA, parity, AUC _glu_, pre-pregnancy BMI, birthweight, child's sex, age, exercise level, and/or height, family history of DM and hypertension as appropriate.

Statistical analyses were performed using SPSS version 25 (SPSS, IBM, Chicago, IL, USA) and R (version 4.0.3) downloadable at www.r-project.org/. P values < 0.05 were used to indicate significance for two-tailed statistical test results.

### 2.3 Role of the funding sources

The HAPO study was funded by the National Institute of Child Health and Human Development (grant no. R01-HD34242) and the National Institute of Diabetes and Digestive and Kidney Diseases (grant no. R01-HD34243). The HAPO follow-up study at the Hong Kong Centre was supported by the funding from the General Research Fund of the Research Grants Council of the Hong Kong SAR, China (grants CUHK 473408 and, in part, CUHK 471713). The funding source had no role in the study design, secondary data analysis, data interpretation or manuscript writing.

## 3 Results

Table 1 showed the maternal characteristics, pregnancy and neonatal outcomes of all eligible 1498 women and the between group comparisons according to their pre-pregnant BMI. In general, underweight mothers were younger and had a lower rate of GDM compared to the other subgroups. They also had a higher rate of delivering lean babies with LBW, while overweight mothers had a relatively higher rate of delivering macrosomic babies who had higher fat mass compared with normal weight mothers. Obese mothers gained significantly less weight than their underweight and normal weight counterparts (p=0.028), with a mean difference of 3.7 and 3.5 kg, respectively.

**Table 1:** Maternal characteristics, pregnancy and neonatal outcomes in the study cohort.

	All	Underweight	Normal weight	Overweight	Obese	P value
	n=1498	n=310	n=1026	n=129	n=33	
Maternal characteristics						
Age (years)	31.0 (27.5-34.2)	29.1 (25.1-32.3)^⁎#^	31.4 (28.1-34.5)*	32.6 (29.0-35.0)^#^	32.0 (27.0-35.2)	<0.001
Nulliparity	910 (60.7)	210 (67.7)*	628 (61.2)^#^	57 (44.2)^⁎#^	15 (45.5)	<0.001
Height (cm)	158 (155-162)	160 (156-163)*	158 (155-162)*	158 (154-162)	158 (154-162)	0.003
Pre-pregnancy BMI (kg/m2)	20.2 (18.8-22.1)	17.6 (17.1-18.1)^*#§^	20.4 (19.5-21.8)^*†§^	25.4 (24.5-26.4)^#†^	29.7 (28.5-31.6) ^§§^	<0.001
GA at first visit (weeks)	11.7 (8.1-14.6)	11.0 (7.7-14.6)	11.0 (8.1-14.6)	10.4 (8.1-13.9)	12.6 (9.6-14.7)	0.645
Smoker ^$^	30 (2.0)	11 (3.5)	16 (1.6)	1 (0.8)	2 (6.1)	0.033
Pregnancy outcomes						
GA at delivery (weeks)	39.6 (38.7-40.4)	39.7 (38.7-40.4)	39.6 (38.7-40.6)	39.4 (38.5-40.4)	39.9 (38.8-40.6)	0.477
GWG (kg)	15.2 (12.4-18.3)	15.5(13.1-18.3)*	15.3 (12.6-18.3)^#^	14.3 (10.3-18.1)	11.8 (8.9-15.4)^⁎#^	<0.001
Primary CS	290 (19.4)	50 (16.1)	203 (19.8)	30 (23.3)	7 (21.2)	0.318
GH/PE ^$^	27 (1.8)	5 (1.6)	21 (2.1)	0	1 (3.0)	0.296
GDM	196 (13.1)	25 (8.06)^⁎#^	143 (14.0)*	22 (17.1)^#^	6 (18.2)	0.018
Neonatal outcomes						
Birth weight (g)	3190 (2935-3465)	3135 (2870-3356)^*#§^	3190 (2950-3470)^⁎†^	3295 (3017.5-3618)^#†^	3395 (3027.5-3615)^§^	<0.001
Sex (male)	783 (52.3)	138 (44.5)*	563 (54.9)*	66 (51.2)	16 (48.5)	0.015
Fat mass‡ (g)	308.7 (229.0-410.4)	279.6 (199.6-371.2)^*#§^	312.5 (235.9-408.6)*	349.2 (242.7-466.4)^#^	415.2 (265.8-458.2)^§^	<0.001
LBW	42 (2.8)	14 (4.5)	22 (2.1)	4 (3.1)	2 (6.1)	0.063
Macrosomia	38 (2.5)	4 (1.3)*	25 (2.4)	8 (6.2)*	1 (3.0)	0.035
SGA	151 (10.1)	45 (14.5)*	92 (9.0)*	9 (7.0)	5 (15.2)	0.015
LGA	103 (6.9)	16 (5.2)*	61 (5.9)^#^	21 (16.3)^⁎#^	5 (15.2)	<0.001
Lean‡	119 (8.5)	35 (12.4)	72 (7.5)	9 (7.5)	3 (9.7)	0.078
Fat‡	146 (10.5)	23 (8.1)*	94 (9.8)^#^	26 (21.7)^⁎#^	3 (9.7)	<0.001

Normally distributed continuous variables were expressed as mean ± SD and compared using one-way ANOVA;

Non-normally distributed continuous variables were expressed as median (IQR) and compared by using Kruskal-Wallis tests;

Categorical data were expressed as n (%) and compared by using Chi-square tests unless otherwise specified ^$^ Fisher's exact test.

Underweight, normal weight, overweight and obese were defined according by maternal pre-pregnant BMI: <18.5, 18.5-23.9, 24.0-27.9 and  ≥28.0 kg/m^2^ respectively.

GA, gestational age; GWG, gestational weight gain; CS, caesarean section; GDM, gestational diabetes mellitus; LBW, low birth weight; SGA, small for gestational age; LGA, large for gestational age; GH, gestational hypertension; PE, pre-eclampsia.

‡ Data on infants’ fat mass were available in 283, 958, 120 and 31 among mothers who were underweight, normal weight, overweight and obese. *#§†§P<0.05 for between group comparison.

With reference to the IOM recommendation, 630 mothers (42.1%) had GWG which exceeded recommendations, whilst 261 (17.4%) had GWG below the recommendations. Meanwhile, we also observed a slightly greater proportion of infants being classified as SGA than being classified as lean (10.1% vs. 8.5%, p=0.04), but a lower rate of infants classified as LGA than being fat (6.9% vs 10.5%, p=0.004) in the entire cohort. The frequency distributions of maternal weight gain of the four subgroups are shown in Supplementary figure 3. Among the underweight mothers, 50.0% had GWG fell within, while 23.5% were below and 26.5 % exceeded the IOM recommendation. Majority of the overweight (63.0%) and obese (76.9%) mothers had GWG exceeding the IOM recommendation; nearly half (44 %) of normal weight mothers also exceeded the IOM recommendation in the weight gain.

Infants’ fat mass and fat-free mass were positively associated with maternal GWG and their own birth weights in a slight curvilinear manner (Supplementary Figure 4), thus the infants’ percentage of body fat increased with maternal GWG and their own birth weights. For each kg increase in maternal GWG, the percentage of body fat (95% CI, p values) increased by 0.30% (0.22-0.38%, p<0.001), 0.15% (0.11-0.20%, p<0.001) and 0.15% (0.03-0.26%, p=0.012) in underweight, normal weight and overweight mothers, respectively. The rate of increase in body fat percentage per kg increase maternal weight gain was 2-fold in children born to underweight mothers compared with those born to normal weight and overweight mothers. On the other hand, for each 100-gram increase in the infants’ birth weight, the rate of increase in body fat percentage (95% CI, p values) were similar in all three subgroups: i.e. 0.70% (0.64-0.76%, p<0.001), 0.66% (0.63-0.69%, p<0.001) and 0.75% (0.67-0.83%, p<0.001) in underweight, normal weight and overweight mothers, respectively.

**Figure 1:**
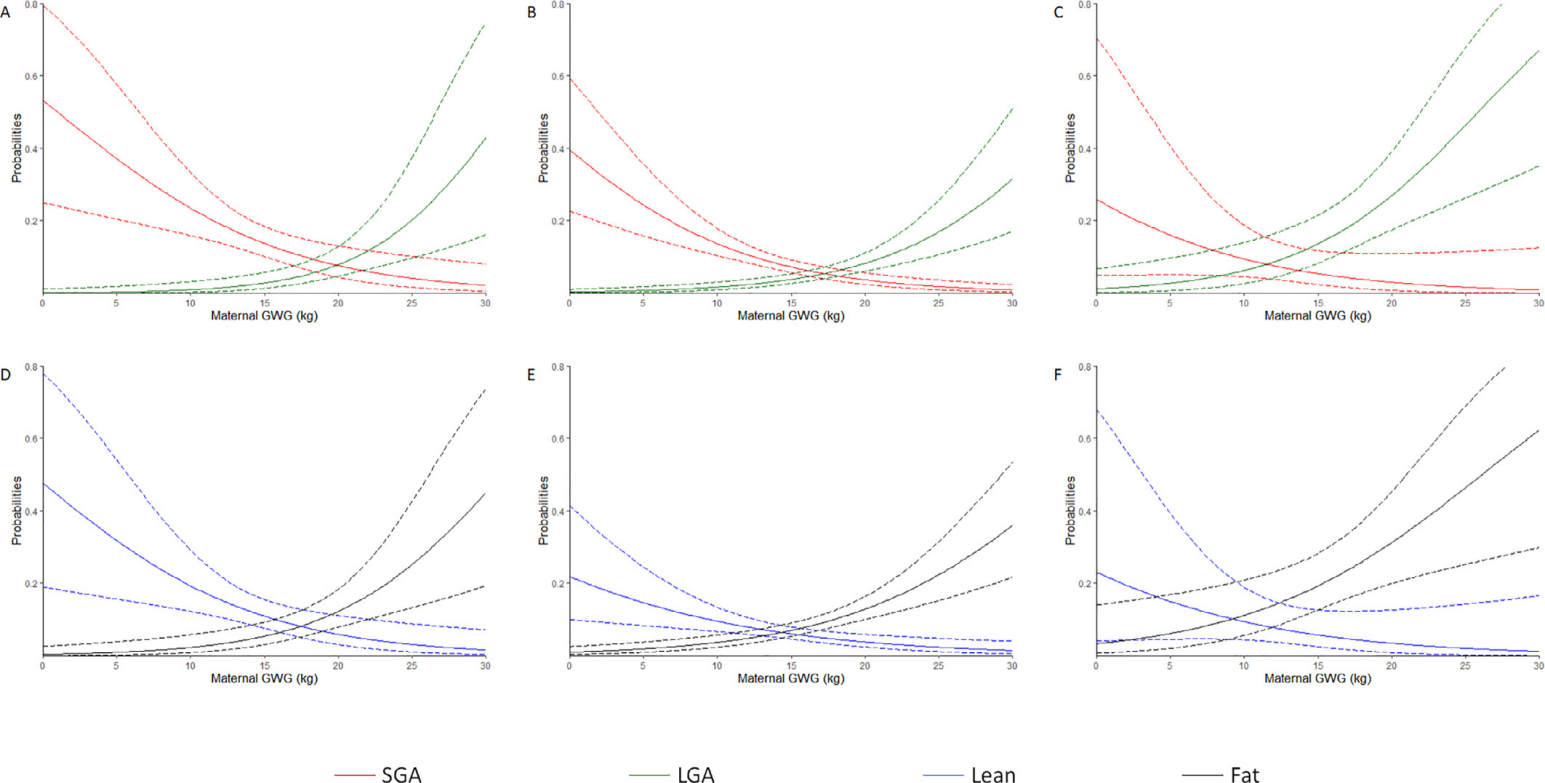
Probabilities of neonatal adverse pregnancy outcomes associated with maternal GWG by logit link. The solid curve represents probabilities of neonatal adverse pregnancy outcomes associated with maternal GWG, and the dashed curve represents 95% confidence interval of the probabilities. (A, B and C) Probabilities of an infant born SGA or LGA in the underweight, normal weight and overweight pre-pregnancy BMI categories, respectively. (D, E and F) Probabilities of an infant born lean or fat in the underweight, normal weight and overweight pre-pregnancy BMI categories, respectively.

Maternal GWG significantly altered the odds of neonates being SGA, LGA, lean and fat at birth in women who were underweight and normal weight after adjustment for various confounders. However, maternal GWG was only found to alter the odds of neonates born LGA and fat after similar adjustment in overweight mothers. The difference of AIC values between logit link and complementary log-log link were all less than 1 (supplementary table 1). These associations obtained by using logit link were also demonstrated in Figure 1 (A-F) showing the probabilities (95% CI) of SGA, LGA, lean and fat at birth in underweight, normal weight and overweight women.

**Figure 2:**
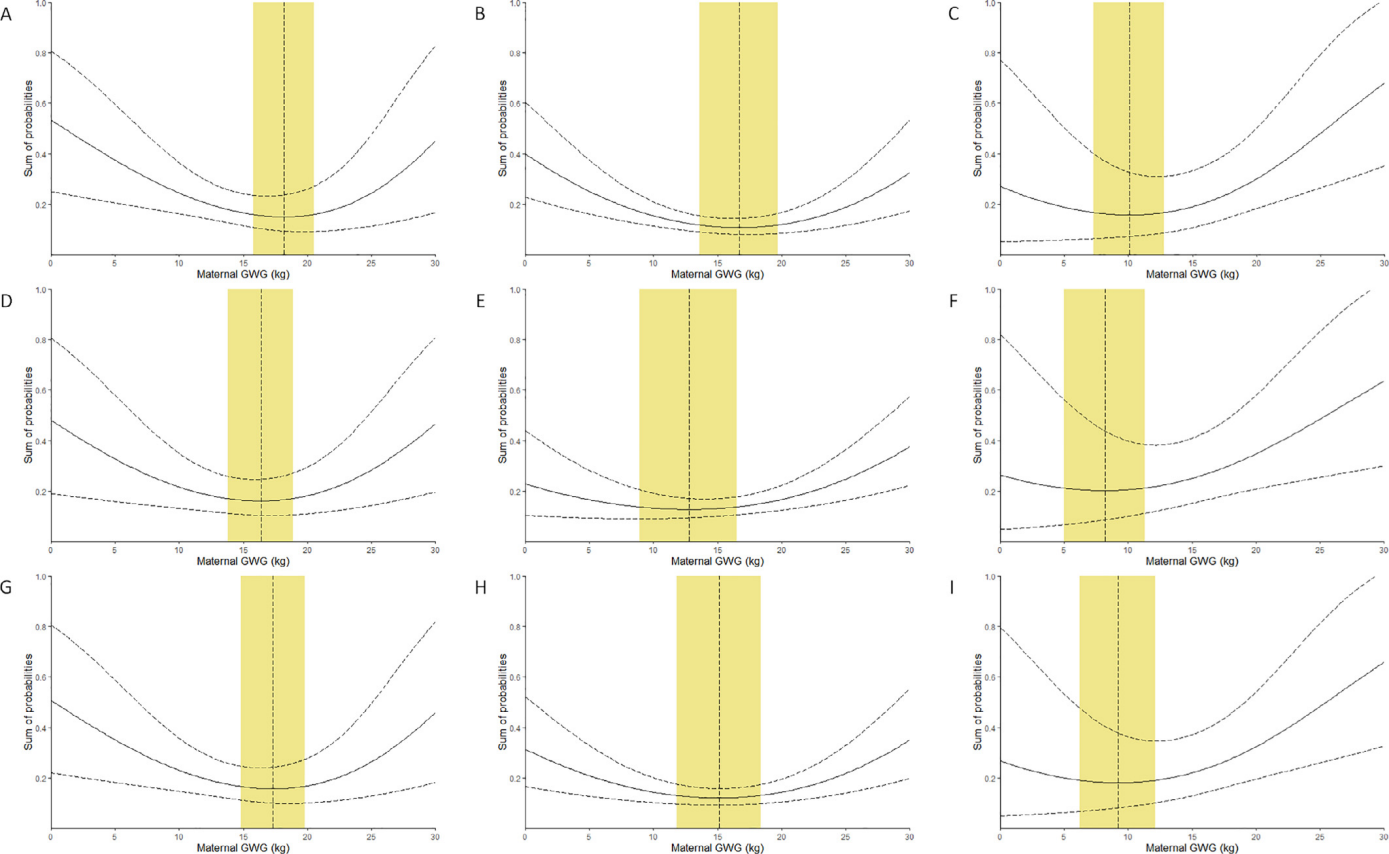
Sum of probabilities in neonatal adverse pregnancy outcomes associated with maternal GWG by logit link The solid curve represents sum of probabilities in neonatal adverse pregnancy outcomes associated with maternal GWG, and the dashed curve represents 95% confidence interval of sum of probabilities. The yellow area represents the reference range for optimal GWG, and the dashed line inside represents the GWG with lowest sum of probabilities. (A, B and C) Sum of probabilities in infants born SGA and LGA (model 1) in the underweight, normal weight and overweight pre-pregnancy BMI categories, respectively. (D, E and F) Sum of probabilities in infants born lean and fat (model 2) in the underweight, normal weight and overweight pre-pregnancy BMI categories, respectively. (G, H and I) Sum of probabilities in infants born SGA, LGA, lean and fat (model 3) in the underweight, normal weight and overweight pre-pregnancy BMI categories, respectively.

The sum of probabilities (95% CI) of SGA and LGA (model 1, derived from equation 3), of lean and fat (model 2, derived from equation 4) and of SGA, LGA, lean and fat (model 3, derived from equation 5) by logit link were shown in Figure 2 (A-C), (D-F) and (G-I) respectively in the 3 pre-pregnancy BMI categories, depicting also the maternal GWG with the lowest sum of probabilities and shaded area representing ranges which bounded the 1% rise in the sum of probabilities adverse outcomes in each model. The details of the optimal GWG were tabulated in supplementary table 2. The logit link performed better in the underweight and overweight groups, whereas the complementary log-log link performed better in the normal weight group, according to the sum of AIC values in each group (supplementary table 1). The reference ranges for the optimal GWG derived from the better link with confined decimals were presented in table 2.

**Table 2:** Reference ranges for optimal gestational weight gain (GWG) derived from the 3 models and their comparisons with the recommendation from the IOM

	Optimal GWG (kg)		
	Underweight	Normal weight	Overweight
Model 1	16.0 – 20.5	14.0 – 19.5	7.5 – 12.5
Model 2	14.0 – 18.5	9.0 – 16.5	5.0 – 11.0
Model 3	15.0 – 19.5	12.0 – 18.5	6.5 – 12.0
IOM	12.5 – 18.0	11.5 – 16.0	7.0 – 11.5

Model 1 was based on the lowest sum of probabilities in SGA and LGA; model 2 was based on the lowest sum of probabilities in fat and lean infants); model 3 was the integration of models 1 & 2 (i.e. the lowest sum of probabilities of SGA, LGA, fat and lean infants).

Underweight, normal weight and overweight according to the model 1 to 3 were defined on the basis of the maternal pre-pregnant BMI: <18.5, 18.5-23.9, 24.0-27.9 kg/m^2^, while those according to the IOM recommendation were defined on the basis of the maternal pre-pregnant BMI: <18.5, 18.5-24.9, 25.0-29.9 kg/m^2^, respectively. IOM, Institute of Medicine.

In contrast, the adjusted ORs (95% CI) of nulliparity associated with SGA, LGA, lean and fat in the overall cohort were 1.4 (0.94-2.1), 0.45 (0.28-0.71), 1.8 (1.2-2.9) and 0.46 (0.31-0.68), respectively. Optimal GWG ranges for the multiparous women appeared to be lower than that for the nulliparous (supplementary table 3), as well as that for all pregnant women regardless of parity (supplementary table 2).

The maternal and neonatal adverse outcomes of mothers who gained weight within the optimal GWG in models 1 to 3 were compared with each other as well as with that recommended by the IOM were shown in table 3. The proportion of those who fell within the recommenced GWG ranges obtained from model 2 and 3 were 50.2% and 49.6%, which were greater than that from model 1 (40.3%) and the IOM (40.8%), p values <0.001. However, there was no significant difference in outcomes amongst the mothers who had GWG at optimal ranges according to model 1 to 3 groups and that recommended by the IOM.

**Table 3:** Maternal and neonatal outcomes among pregnancy with GWG within optimal range derived from models 1 to 3 and that of IOM.

	Overall (n=1465)	Model 1 (n=590)	Model 2 (n=738)	Model 3 (n=726)	IOM (n=597)	P value
Maternal outcomes						
Primary CS	283 (19.3)	117 (19.8)	134 (18.2)	154 (21.2)	121 (20.3)	0.524
Gestational hypertension/pre-eclampsia	26 (1.8)	15 (2.5)	12 (1.6)	18 (2.5)	10 (1.7)	0.490
GDM	190 (13.0)	79 (13.4)	95 (12.9)	97 (13.4)	86 (14.4)	0.877
Neonatal outcomes						
Hypoglycaemia	66 (4.5)	24 (4.1)	32 (4.3)	31 (4.3)	30 (5.0)	0.863
LBW	40 (2.7)	8 (1.4)	23 (3.1)	18 (2.5)	19 (3.2)	0.152
Macrosomia	37 (2.5)	9 (1.5)	12 (1.6)	11 (1.5)	11 (1.8)	0.966
SGA	146 (10.0)	41 (6.9)	77 (10.4)	55 (7.6)	58 (9.7)	0.072
LGA	98 (6.7)	39 (6.6)	32 (4.3)	39 (5.4)	27 (4.5)	0.249
Lean*	116 (8.5)	38 (6.9)	66 (9.7)	50 (7.4)	48 (8.8)	0.252
Fat*	143 (10.5)	60 (10.8)	58 (8.5)	66 (9.8)	45 (8.2)	0.398

Data were expressed as n (%) and compared by using Chi-square tests.

⁎ Data on fat mass were available in 554, 683, 676 and 546 infants for model 1, model 2, model 3 and the IOM only. Model 1 was based on the lowest sum of probabilities in SGA and LGA; model 2 was based on the lowest sum of probabilities in fat and lean infants); model 3 was the integration of models 1 & 2 (i.e. the lowest sum of probabilities of SGA, LGA, fat and lean infants). Underweight, normal weight and overweight were defined according to the maternal pre-pregnant BMI: <18.5, 18.5-23.9, 24.0-27.9 kg/m^2^ respectively. CS, caesarean section; GDM, gestational diabetes mellitus; LBW, low birth weight; SGA, small for gestational age; LGA, large for gestational age; IOM, Institute of Medicine.

Table 4 compared the associations of children's cardiometabolic risks at seven years of age with different GWG ranges from model 1 to 3 and that recommended by IOM. After excluding 23 children who were born to obese mothers, data on 882 children were analysed.

**Table 4:** Associations between different references for maternal GWG and offspring cardiometabolic risk factors at 7 years of age

Long-term risks	GWG below the references												GWG exceeding the references										
	Model 1			Model 2			Model 3			IOM			Model 1			Model 2			Model 3			IOM	
	n=347 (39.3)			n=88 (10.0)			n=210 (23.8)			n=152 (17.2)			n=181 (20.5)			n=329 (37.3)			n=224 (25.4)			n=358 (40.6)	
	Coeffecients			Coeffecients			Coeffecients			Coeffecients			Coeffecients			Coeffecients			Coeffecients			Coeffecients	
Adiposity traits																							
BMI (kg/m2)	-0.25(-0.58 to 0.07)			-0.30(-0.81 to 0.20)			-0.30(-0.66 to 0.05)			-0.01(-0.42 to 0.40)			0.42(0.03 to 0.82)*			0.54(0.22 to 0.85)⁎⁎⁎⁎			0.58(0.22 to 0.93)⁎⁎⁎			0.54(0.23 to 0.86)⁎⁎⁎	
WC (cm)	-0.56(-1.3 to 0.22)			-1.1(-2.3 to 0.10)			-0.68(-1.5 to 0.18)			-0.47(-1.5 to 0.52)			0.74(-0.21 to 1.7)			0.85(0.10 to 1.6)*			0.90(0.06 to 1.8)*			0.81(0.04 to 1.6)*	
SSF thickness (mm)	-1.4(-3.8 to 0.92)			-1.8(-5.6 to 1.8)			-1.3(-3.9 to 1.3)			-0.55(-3.6 to 2.5)			2.6(-0.31 to 5.5)			2.0(-0.34 to 4.3)			3.5(0.89 to 6.1)⁎⁎			2.0(-0.32 to 4.4)	
Blood pressure																							
SBP (mmHg)	-0.50(-1.7 to 0.74)			-0.66(-2.6 to 1.3)			-0.28(-1.6 to 1.1)			-0.11(-1.7 to 1.5)			1.3(-0.20 to 2.8)			0.95(-0.25 to 2.2)			1.9(0.56 to 3.3)⁎⁎			0.99(-0.23 to 2.2)	
DBP (mmHg)	-0.49(-1.6 to 0.68)			-0.31(-2.1 to 1.5)			-0.02(-1.3 to 1.3)			1.1(-0.40 to 2.5)			1.4(-0.10 to 2.7)			1.5(0.35 to 2.6)⁎⁎			1.7(0.41 to 2.9)⁎⁎			1.6(0.50 to 2.8)⁎⁎	
Glucose and insulin levels																							
FPG (mmol/l)	0.02(-0.04 to 0.07)			0.04(-0.05 to 0.12)			-0.001(-0.06 to 0.06)			-0.04(-0.11 to 0.03)			0.04(-0.04 to 0.09)			-0.001(-0.05 to 0.05)			0.02(-0.04 to 0.08)			-0.03(-0.09 to 0.02)	
2 h glucose(mmol/l)	0.09(-0.06 to 0.24)			0.11(-0.12 to 0.34)			0.03(-0.13 to 0.20)			-0.002(-0.19 to 0.19)			-0.09(-0.27 to 0.09)			-0.05(-0.20 to 0.09)			-0.10(-0.26 to 0.06)			-0.08(-0.22 to 0.07)	
FPI (pmol/l)	-0.46(-1.0 to 0.10)			-0.22(-1.1 to 0.64)			-0.28(-0.90 to 0.34)			0.34(-0.37 to 1.0)			0.34(-0.34 to 1.0)			0.63(0.09 to 1.2)*			0.39(-0.22 to 1.0)			0.80(0.25 to 1.4)⁎⁎⁎	
2 h insulin(pmol/l)	-1.2(-5.0 to 2.6)			1.6(-4.3 to 7.5)			0.38(-3.8 to 4.6)			2.9(-1.8 to 7.7)			3.7(-0.94 to 8.3)			4.4(0.77 to 8.1)*			3.8(0.37 to 7.9)			4.6(0.88 to 8.4)*	
Beta cell function and insulin sensitivity																							
HOMA-β	-6.9(-18.1 to 4.3)			0.89(-16.4 to 18.2)			-0.39(-12.8 to 12.0)			12.3(-1.8 to 26.4)			6.3(-7.4 to 20.0)			12.7(1.9 to 23.5)*			5.1(-7.1 to 17.3)			17.7(6.7 to 28.7)⁎⁎⁎	
IGI at 30 min	-7.8(-22.2 to 6.7)			9.4(-13.0 to 31.7)			-1.1(-17.1 to 15.0)			13.3(-4.9 to 31.5)			11.4(-6.3 to 29.0)			16.4(2.4 to 30.4)*			16.2(-0.40 to 31.9)*			20.1(5.8 to 34.3)⁎⁎	
Matsuda ISI	0.58(-0.81 to 2.0)			-0.45(-2.6 to 1.7)			-0.47(-2.0 to 1.1)			-1.7(-3.5 to -0.02)			-1.6(-3.3 to 0.15)			-1.6(-3.0 to -0.29)*			-0.20(-3.5 to -0.49)⁎⁎			-2.1(-3.4 to -0.70)⁎⁎⁎	
ODI	0.18 (-1.3 to 1.6)			1.6 (-0.63 to 3.9)			0.29 (-1.3 to 1.9)			1.0 (-0.82 to 2.9)			-0.32 (-2.1 to 1.5)			0.37 (-1.0 to 1.8)			-0.22 (-1.8 to 1.4)			0.45 (-0.98 to 1.9)	

Data are expressed as coeffecient (95% CI).

Model 1: reference determined by birth weight (with the lowest sum of probabilities in SGA and LGA); model 2: reference determined by adiposity (with the lowest sum of probabilities in fat and lean infants); model 3: reference determined by integrating model 1 & 2 (the lowest sum of probabilities of SGA, LGA, fat and lean infants).

Coefficients were adjusted for maternal pre-pregnant BMI, parity, maternal age, gestational age at delivery, maternal AUC_glu_ during pregnancy, neonatal birthweight and sex, age and exercise level at childhood; further adjusted for childhood height for waist circumference and sum of skinfold thickness; current maternal hypertensive status and childhood height for blood pressure; or further adjusted for current maternal and paternal diabetes status for glucose and insulin levels, as well as indices for beta cell function and insulin sensitivity.

GWG, gestational weight gain; IOM, Institute of Medicine; WC, waist circumference; SSF, sum of skinfold, SBP and DBP, systolic and diastolic blood pressure; FPG and FPI, fasting plasma glucose and insulin; HOMA-β, homeostasis model assessment of beta cell function; IGI, insulinogenic index; ISI, insulin sensitivity index; ODI, oral disposition index.

⁎ 0.01≤ P< 0.05;

⁎⁎ 0.005≤ P< 0.01;

⁎⁎⁎ 0.001≤ P< 0.005;

⁎⁎⁎⁎ P< 0.001.

Model 1 was found to be only associated with children's BMI, whereas the other two models and that from IOM were associated with children's adiposity traits, blood pressure, beta-cell function and insulin sensitivity to different degree when the mothers gained weight exceeding the optimal ranges and the respective recommendation. Compared to model 3, children whose mother has GWG exceeding reference range derived from model 2 were associated with more insulin indices (namely FPI, 2h insulin, HOMA-β, IGI and Matsuda ISI).

## 4 Discussion

The main objective of our study is to explore the association between maternal GWG in pregnancy with adverse pregnancy outcomes and to determine the appropriate weight gain reference, which would result in optimal infant body size and fat mass, along with other optimal pregnancy outcomes. From the three models that are based on the infants’ birth weight, their adiposity or the combined model using both birth weight and adiposity together, models based on adiposity appears most discriminative. The model is better at predicting the children's future cardiometabolic risk, predominantly their adiposity measures, blood pressure, insulin resistance and pancreatic beta cell secretory function.

Contrary to the widely accepted IOM recommendation for the Caucasian population, there has never been a similar universally agreed recommendation for the Asian population. All the recommendations were mostly based on the infants’ rates of SGA and LGA, and other adverse pregnancy outcomes such as preterm delivery, caesarean section, GDM and preeclampsia, but seldom touched on other neonatal complications, nor the children's long-term health. Taking into consideration several recent studies showing the influence of GWG on the children's birth weight and their cardiometabolic health, we also included the children's cardiometabolic traits in the exploration of optimal weight gain in pregnancy. [3,12,21]

The main disadvantage of modelling GWG reference ranges solely based on SGA and LGA, which are derived from infant's birth weight alone, is the inability to differentiate between the infants’ fat mass, i.e. their adiposity, from their fat-free mass. In our study, we observed a higher rate of infants being classified as lean rather than SGA, but a lower rate of infants classified as fat than being LGA. As neonatal adiposity could be reliably estimated from routine anthropometric measurements at birth, it is now feasible and more practical to include adiposity into the outcome in future studies which aim to establish the optimal GWG. [22]

Sparks proposed that the *in utero* environment contributed mainly to the infant's fat mass, whereas lean body mass or fat-free mass was the genetic component of foetal growth. [23] Our study finding confirmed that children's percentage of fat mass increased with maternal GWG. Similarly, the Healthy Start study also demonstrated that infants increased differentially in their fat mass, fat-free mass and hence percentage of body fat, with excessive maternal GWG. [10] Other studies also consistently showed that higher maternal fat and carbohydrate intake, as well as inadequate physical activities in the mid and late gestations were associated with greater neonatal fat mass, but not neonatal fat-free mass and birth weight. [24,25]

The median GWG in all four BMI subgroups of our cohort were comparable with that reported in two other Chinese populations from urban Shanghai and Changsha. [4,26] However, GWG among underweight and normal weight mothers in Wuhan were in average 3.5 kg and 2 kg greater than ours and the other two cohorts. [27] On the other hand, our study cohort also had a much lower rate of macrosomia (2.5%) than other Chinese cohorts (6.1-8.6%). [4,27,28]

Similar to that suggested from a Dutch study, [29] our findings suggested that the upper reference of GWG in women, for women who are underweight with BMI <18.5 kg/m^2^, could be 0.5-1.5kg greater than that currently recommended by the IOM. For normal weight and overweight, the upper reference levels are similar to that of IOM. Compared to the IOM recommendation, the reference range for GWG determined from neonatal adiposity in the present study provides a wider interval, hence re-classifying 10% of the mothers initially outside the recommended reference based on the IOM, into within recommended GWG according to the proposed reference. It has been reported earlier that in a multi-ethnic population, overweight and obese women gaining less than 5 kg in pregnancy will have higher risk of SGA, decreased neonatal fat mass, lean mass and head circumference. [30] The lower reference of optimal GWG in the overweight mothers was 5 kg in the present study.

Additionally, parity was found to be associated with the risks of infants born LGA, lean or fat. Optimal GWG ranges for multiparous women appeared to be lower than for the whole population. The present recommendations, including that from the IOM, had not considered the impact of parity separately. Further study with a larger population size are required to develop separate recommendations for nulliparous and multiparous women.

The present study was however limited by the small sample size, and inadequate number of obese mothers with pre-pregnancy BMI > 28 kg/m^2^ for any subgroup analysis. Mothers recruited into the original HAPO study were healthy without previous history of hyperglycaemia and relatively young. Therefore, the cohort may not be entirely representative of the general obstetric population, which would involve a broader range of BMI, as well as including mothers with medical conditions. Moreover, there is no good standard reference on the estimation of neonatal fat mass. Nonetheless, the computation of fat mass from the skinfold thickness had been used for several times in the previous publications with the other HAPO study papers. [11,17] Similar to previous studies on gestational weight gain, there is no unified reference on how to calculate the total weight gain in pregnancy. Previous study has used the maternal weight at 37 weeks as the endpoint to estimate GWG. [29] Alternatively, we can also estimate the weight at 40 weeks of gestation as the endpoint for the calculation. In the present study, we used maternal weight at the time of just prior to delivery as the endpoint.

## 5 Conclusion

The present study highlighted the methodology to assess an optimal GWG in Chinese women during pregnancy and compared that with the recommendation from IOM. The standard birth weight varies among different ethnic groups so as the definition of SGA and LGA. The baby's fat mass and fat-free mass increase at a slightly different rate with baby's own birth weight and mother's GWG. We could demonstrate in the present study the optimal GWG derived is associated with lower rate of lean and fat babies in underweight, normal weight and overweight Chinese mothers.

## 6 Authors’ Contribution

This study was conceptualized and designed by Dr Wing Hung Tam. Dr Claudia H.T. Tam contributed to the study design, methodology and advised on the statistical analysis. Dr Yuanying He performed all the data analysis and wrote the manuscript. Dr Wing Hung Tam and Dr Ronald C.W. Ma were responsible for obtaining funding, and acquired all the data from the HAPO follow-up study with Ms Lai Yuk Yuen. Dr Patrick M. Catalano contributed to the study design and advised on the data analysis. All authors contributed to the critical review of the manuscript.

## 7 Data sharing statement

**Table:** 

Will individual participant data be available (including data dictionaries)?	Yes
What data in particular will be shared?	Individual participant data that underlie the results reported in this article, after deidentification (text, tables, figure).
What other documents will be available?	Study Protocol
When will data be available (start and end dates)?	Beginning 9 months and ending 36 months following article publication.
With whom?	Investigators whose proposed use of the data has been approved by an independent review committee identified for this purpose.
For what types of analyses?	For individual participant data meta-analysis.
By what mechanism will data be made available?	Proposals should be directed to: tamwh@cuhk.edu.hk. Approval from the HAPO study steering committee should be obtained before the sharing of the original HAPO study data. To gain access, data requestors will need to sign a data access agreement.

## Declaration of Competing Interest

All the authors declared no conflict of interest.

## Appendix Supplementary materials

Image, application 1

Image, application 2
