# Supplementary material for: Optimal gestational weight gain for Chinese women - analysis from a longitudinal cohort with childhood follow-up
Source: Lancet Reg Health West Pac. 2021 Jul 6;13:100190. doi: 10.1016/j.lanwpc.2021.100190 (PMC8403904; doi:10.1016/j.lanwpc.2021.100190)
Supplement: Supplementary file 2 [file mmc2.docx]

**Supplementary note 1: Equations for the estimation of probabilities of adverse outcomes associated with maternal GWG in Figure 1 and Figure 2**

a. Individual probability:

Equation 1 and 2 were used to estimate the probability of adverse pregnancy outcomes for each pre-pregnant BMI subgroup by using “logit” and “complementary log-log” links, respectively.

Equation 1:

Probability = $\frac{exp(\beta_{0}+\beta_{1}GWG+\beta_{2}\bar{x}_{2}{+\beta}_{3}\bar{x}_{3}+\beta_{4}\bar{x}_{4})}{1+exp(\beta_{0}+\beta_{1}GWG+\beta_{2}\bar{x}_{2}{+\beta}_{3}\bar{x}_{3}+\beta_{4}\bar{x}_{4})}$

Equation 2:

Probability = 1-exp [-exp ($\beta_{0}+\beta_{1}GWG+\beta_{2}\bar{x}_{2}{+\beta}_{3}\bar{x}_{3}+\beta_{4}\bar{x}_{4}$)]

The coefficients of individual probability (β) were derived through binary regression models by these two links, using SGA, LGA, being lean or fat at birth as dependent variables, and maternal GWG as independent variable, adjusting for maternal age, parity, AUC_glu_, gestational hypertension/ preeclampsia and smoking status. β_0_ to β_4_ represent the estimated coefficients: 0=constant, 1=GWG, 2=maternal age, 3=parity, 4= AUC_glu_, respectively. $\bar{x}$_2_ to $\bar{x}$_4_ represent the means of maternal age, parity and AUC_glu_.

The probability of each dependent variable was associated with each 0.1 kg increment of maternal GWG from 0 to 30 kg.

(GWG, gestational weight gain; SGA, small for gestational age; LGA, large for gestational age; AUC_glu_, area under the curve of glucose level during OGTT.)

b. Sum of probabilities

Equation 3 to 5 is used to estimate the sum of probabilities according to different models

Equation 3:

Sum of probabilities in model 1 *=* $\left( Probability of SGA \right)+(Probability of LGA)$

Equation 4:

Sum of probabilities in model 2 *=* $\left( Probability of being lean \right)+\left( Probability of being fat \right)$

Equation 5:

Sum of probabilities in model 3 $=\frac{\left( Sum of probabilities in model 1 \right)+(Sum of probabilities in model 2)}{2}$

**Supplementary table 1. The risk of SGA, LGA, lean and fat infant in association with maternal GWG for underweight, normal weight and overweight mothers by different links**

|  | Logit |  |  | Complementary log-log | | |
| --- | --- | --- | --- | --- | --- | --- |
|  | Adjusted OR | P | AIC | Adjusted OR | P | AIC |
| Underweight |  |  |  |  |  |  |
| SGA | 0.88 (0.81-0.95) | 0.002 | 254.5 | 0.89 (0.83-0.96) | 0.002 | 254.6 |
| LGA | 1.25 (1.11-1.40) | <0.001 | 121.6 | 1.21 (1.09-1.35) | <0.001 | 122.4 |
| Lean | 0.88 (0.80-0.96) | 0.005 | 211.8 | 0.89 (0.82-0.96) | 0.005 | 212.0 |
| Fat | 1.19 (1.08-1.32) | <0.001 | 157.4 | 1.18 (1.08-1.29) | <0.001 | 157.6 |
| Normal weight |  |  |  |  |  |  |
| SGA | 0.87 (0.82-0.92) | <0.001 | 583.6 | 0.87 (0.83-0.92) | <0.001 | 582.9 |
| LGA | 1.18 (1.11-1.25) | <0.001 | 424.9 | 1.16 (1.10-1.23) | <0.001 | 424.7 |
| Lean | 0.91 (0.85-0.97) | 0.002 | 489.2 | 0.91 (0.86-0.97) | 0.002 | 489.0 |
| Fat | 1.14 (1.08-1.21) | <0.001 | 571.1 | 1.13 (1.08-1.18) | <0.001 | 571.3 |
| Overweight |  |  |  |  |  |  |
| SGA | 0.89 (0.77-1.03) | 0.108 | 71.1 | 0.89 (0.78-1.02) | 0.103 | 71.1 |
| LGA | 1.19 (1.08-1.31) | <0.001 | 110.9 | 1.16 (1.07-1.26) | <0.001 | 110.9 |
| Lean | 0.90 (0.78-1.04) | 0.164 | 70.0 | 0.90 (0.79-1.04) | 0.158 | 69.9 |
| Fat | 1.14 (1.04-1.25) | 0.006 | 119.2 | 1.11 (1.04-1.20) | 0.003 | 119.4 |

Data are expressed as OR (95% CI). ORs were calculated by using “logit” and “complementary log-log” links, and were adjusted for maternal age, gestational age at birth, parity, gestational hypertension/pre-eclampsia, smoking in pregnancy (yes/no), glucose level (area under the curve of glucose levels at 0, 60 and120 min at OGTT between 24-32 weeks) and baby’s gender. Underweight, normal weight and overweight were defined according to the maternal pre-pregnant BMI: <18.5, 18.5-23.9, 24.0-27.9 kg/m^2^ respectively.

OR, odds ratio; AIC, Akaike information criterion; GWG, gestational weight gain; SGA, small for gestational age; LGA, large for gestational age.

**Supplementary table 2. Optimal gestational weight gain (GWG) derived from the 3 models by different links**

|  | Optimal GWG (kg) | | | | | |
| --- | --- | --- | --- | --- | --- | --- |
|  | Logit | | | Complementary log-log | | |
|  | With lowest risks | Lower bounds | Upper bounds | With lowest risks | Lower bounds | Upper bounds |
| Underweight |  |  |  |  |  |  |
| Model 1 | 18.2 | 15.7 | 20.5 | 18.4 | 15.8 | 20.8 |
| Model 2 | 16.4 | 13.8 | 18.9 | 16.4 | 13.7 | 18.9 |
| Model 3 | 17.3 | 14.8 | 19.8 | 17.4 | 14.7 | 19.9 |
| Normal weight | |  |  |  |  |  |
| Model 1 | 16.7 | 13.6 | 19.7 | 16.8 | 13.7 | 19.9 |
| Model 2 | 12.8 | 8.9 | 16.5 | 12.9 | 9.0 | 16.7 |
| Model 3 | 15.1 | 11.8 | 18.4 | 15.2 | 11.9 | 18.6 |
| Overweight |  |  |  |  |  |  |
| Model 1 | 10.1 | 7.3 | 12.8 | 10.3 | 7.4 | 13.1 |
| Model 2 | 8.2 | 5.0 | 11.3 | 8.6 | 5.4 | 11.8 |
| Model 3 | 9.2 | 6.2 | 12.1 | 9.6 | 6.5 | 12.5 |

Risks of infants born SGA, LGA, lean or fat were calculated by “logit” and “complementary log-log” links, respectively. Model 1 was based on the lowest sum of probabilities in SGA and LGA; model 2 was based on the lowest sum of probabilities in fat and lean infants); model 3 was the integration of models 1 & 2 (i.e. the lowest sum of probabilities of SGA, LGA, fat and lean infants). The lower and upper bounds were the points at which 1% increase in the lowest risks.

**Supplementary table 3. Optimal gestational weight gain (GWG) derived from the 3 models for nulliparity and multiparity**

|  | Optimal GWG (kg) | | | | | |
| --- | --- | --- | --- | --- | --- | --- |
|  | Nulliparity | | | Multiparity | | |
|  | With lowest risks | Lower bounds | Upper bounds | With lowest risks | Lower bounds | Upper bounds |
| Underweight |  |  |  |  |  |  |
| Model 1 | 19.0 | 16.3 | 21.6 | 17.4 | 15.3 | 19.4 |
| Model 2 | 17.5 | 14.9 | 20.0 | 14.4 | 11.9 | 16.9 |
| Model 3 | 18.2 | 15.5 | 20.8 | 16.2 | 13.9 | 18.4 |
| Normal weight | |  |  |  |  |  |
| Model 1 | 19.0 | 16.0 | 22.0 | 14.2 | 11.1 | 17.2 |
| Model 2 | 15.4 | 11.7 | 19.1 | 9.5 | 5.5 | 13.3 |
| Model 3 | 17.6 | 14.3 | 20.8 | 12.3 | 8.9 | 15.6 |
| Overweight |  |  |  |  |  |  |
| Model 1 | 13.6 | 10.9 | 16.1 | 9.8 | 7.1 | 12.4 |
| Model 2 | 14.1 | 10.9 | 17.3 | 6.4 | 3.4 | 9.2 |
| Model 3 | 13.8 | 10.9 | 16.6 | 8.2 | 5.4 | 11.0 |

Parity (nulliparity vs multiparity) was regarded as a dichotomous covariate in the logistical regression model..

**Supplementary Figure 1 Flow chart**

**
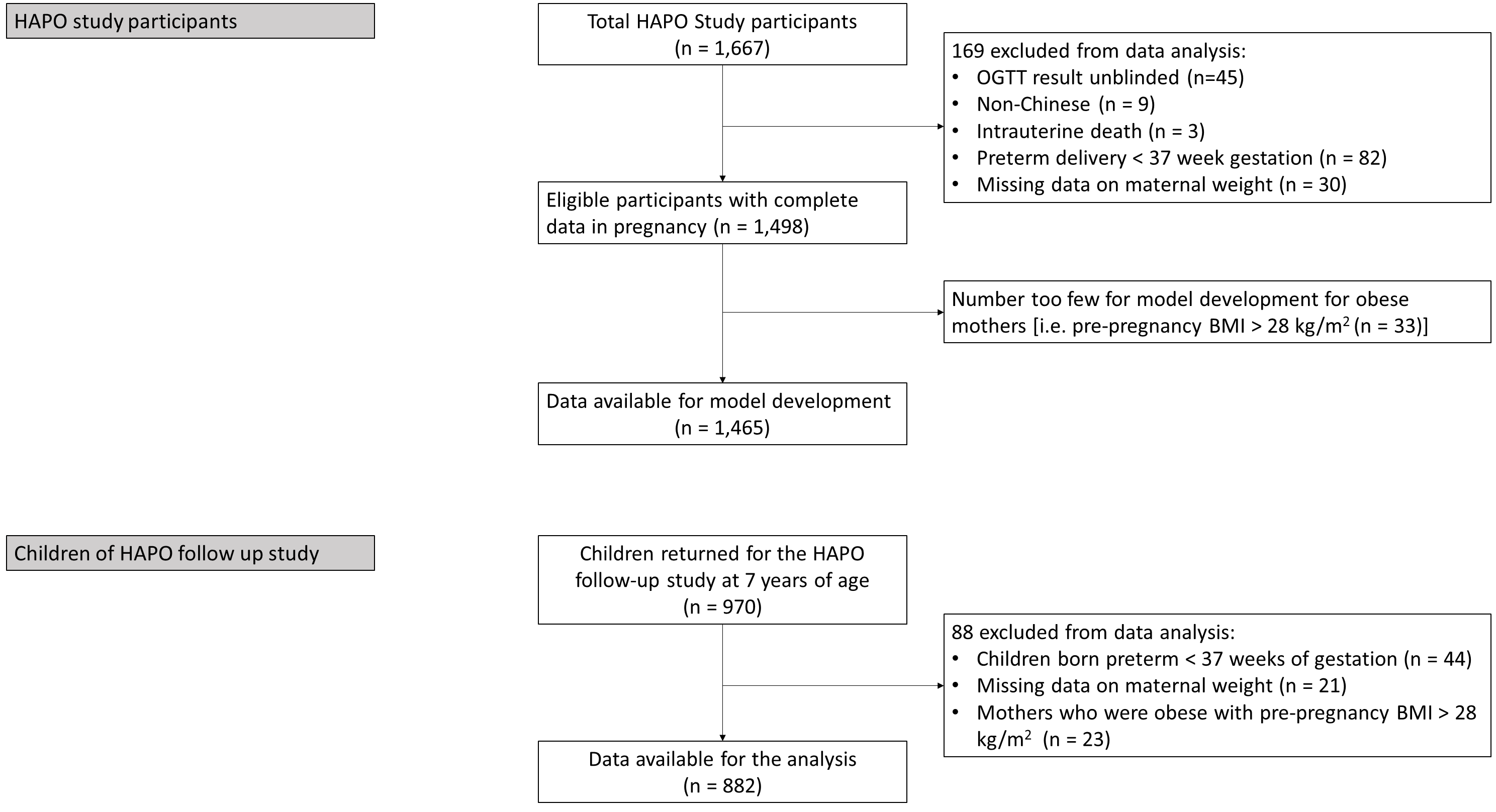
**

**Supplementary Figure 2 Diagram to illustrate the method to derive the optimal gestational weight gain (GWG)**

**
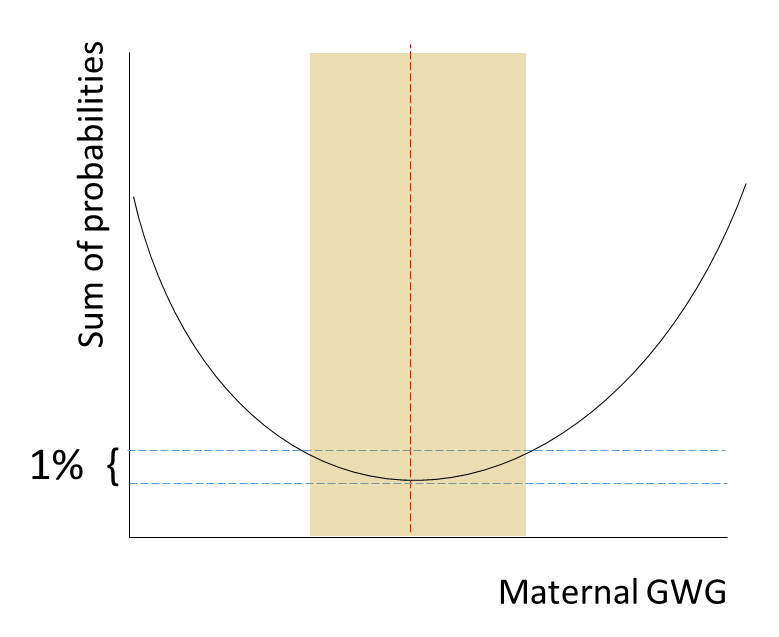
**

Sum of probabilities refer to the sum probabilities of infants born SGA and those born LGA (model 1), infants who were lean and those who were fat at birth (model 2), and the average of both the sum of probabilities in model 1 and model 2 (model 3). The red dashed line indicates the maternal GWG that is associated with the lowest sum of probabilities. The lower and upper bounds of the optimal GWGs, which highlighted by coloured area, were determined at the points which we expect 1% increase in the sum of probabilities.

**Supplementary Figure 3**


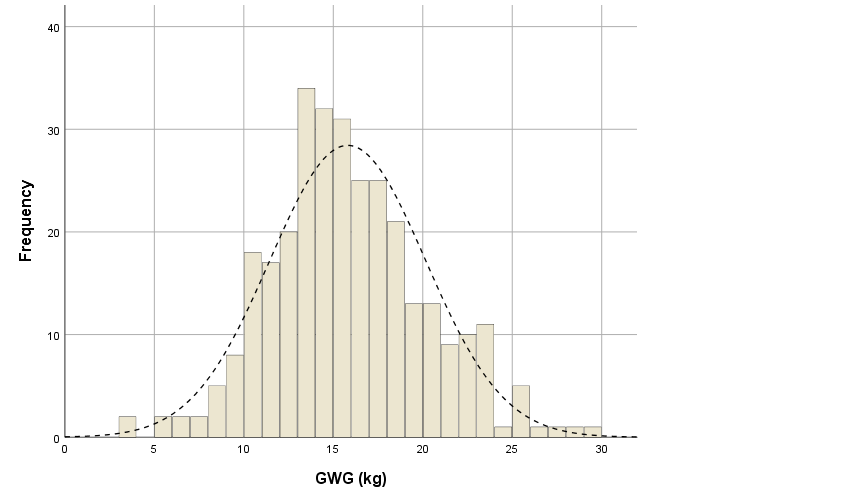


(a)


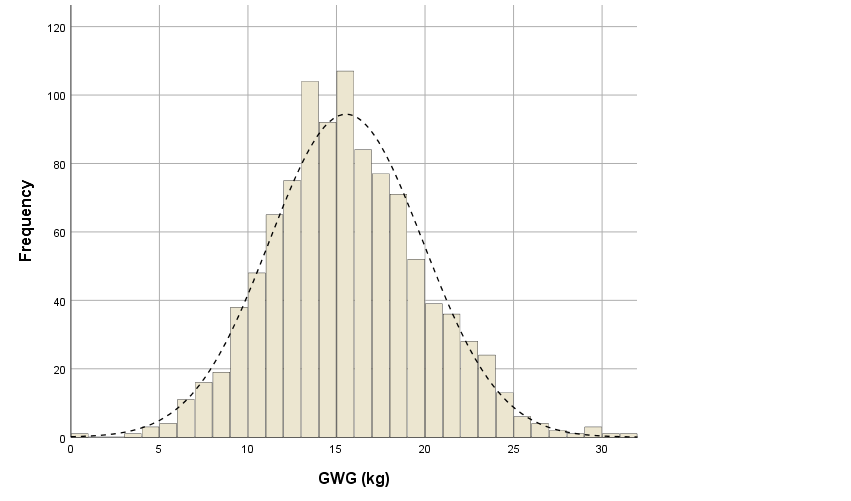


(b)


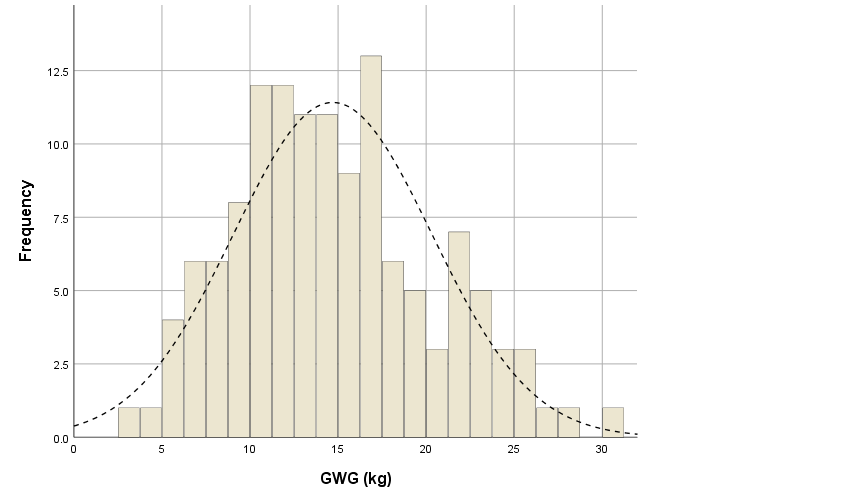


(c)


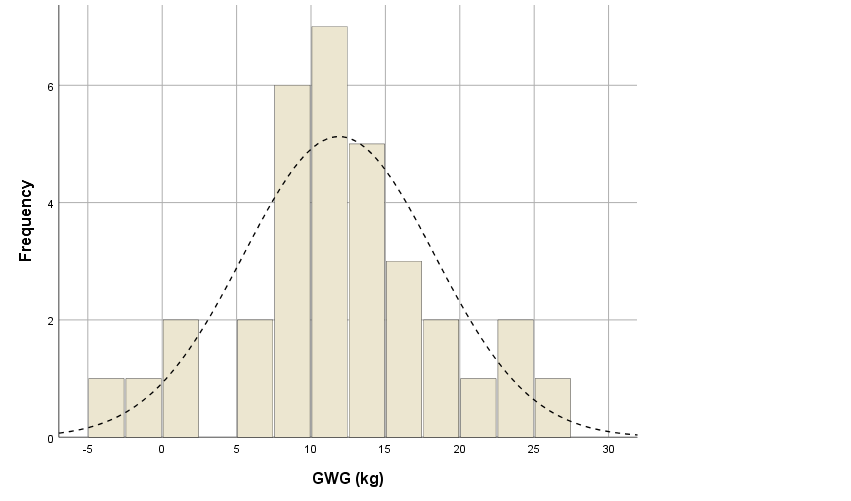


(d)

The frequency distribution of maternal gestational weight gain (GWG) in the HAPO study cohort in the subgroup of mothers who are (a) underweight, (b) normal weight, (c) overweight and (d) obese, based on the maternal pre-pregnant BMI: <18.5, 18.5-23.9, 24.0-27.9 and ≥ 28.0 kg/m^2^ respectively.

**Supplementary Figure 4 Neonatal body compositions in association with birth weight and maternal GWG**

 The orange and grey area represents neonatal fat-free mass and fat mass, respectively, while the dashed line represents neonatal body fat percentage. (A, B and C) Neonatal body compositions associated with birth weight in the underweight, normal weight and overweight pre-pregnancy categories, respectively. (D, E and F) Neonatal body compositions associated with maternal GWG in the underweight, normal weight and overweight pre-pregnancy categories, respectively.
